# Digoxin Enhances the Anticancer Effect on Non-Small Cell Lung Cancer While Reducing the Cardiotoxicity of Adriamycin

**DOI:** 10.3389/fphar.2020.00186

**Published:** 2020-02-28

**Authors:** Yingying Wang, Qian Ma, Shaolu Zhang, Hongyan Liu, Baoquan Zhao, Bo Du, Wei Wang, Peng Lin, Zhe Zhang, Yuxu Zhong, Dexin Kong

**Affiliations:** ^1^Tianjin Key Laboratory on Technologies Enabling Development of Clinical Therapeutics and Diagnostics, School of Pharmaceutical Sciences, Tianjin Medical University, Tianjin, China; ^2^State Key Laboratory of Toxicology and Medical Countermeasures, Beijing Institute of Pharmacology and Toxicology, Beijing, China; ^3^Tianjin Key Laboratory of Biomedical Materials, Institute of Biomedical Engineering, Chinese Academy of Medical Sciences & Peking Union Medical College, Tianjin, China; ^4^Department of Otorhinolaryngology Head and Neck, Institute of Otorhinolaryngology, Tianjin First Central Hospital, Tianjin, China

**Keywords:** digoxin, DNA damage repair, adriamycin, non-small cell lung cancer, decreased cardiotoxicity

## Abstract

Digoxin is widely used to treat heart failure. Epidemiological studies suggested it might be used as an anticancer drug or sensitizing agent for cancer therapy. Adriamycin is a well-known anticancer drug, but often causes cardiotoxicity which limits its use. We recently investigated the anticancer effects of digoxin alone or in combination with adriamycin on human non-small cell lung cancer *in vitro* and *in vivo*. Digoxin reduced the viability of A549 and H1299 cells *in vitro*, increased DNA damage by promoting ROS generation and inhibiting both DNA double strand break (DSB) and single strand break (SSB) repair. Combination with adriamycin showed synergistic antiproliferative effects at the ratios of 1/2IC50_DIG_:IC50_ADR_ and IC50_DIG_:IC50_ADR_ on A549 and H1299 cells, respectively. *In vivo*, digoxin potently inhibited A549 growth in both zebraﬁsh and nude mouse xenograft model. Co-treatment with adriamycin not only enhanced the antitumor efficacy, but also reduced the cardiotoxicity. Our findings suggest that digoxin has the potential to be applied as an antitumor drug *via* inhibiting both DNA DSB and SSB repair, and combination with adriamycin for therapy of human non-small cell lung cancer is reasonable.

## Introduction

Digoxin, as a member of cardiac glycosides, has been in clinical use for the treatment of heart failure and atrial arrhythmias for many years. Several epidemiological studies have shown that patients treated with digoxin developed more benign forms of breast tumors (Stenkvist et al., 1979; Adams et al., 2005). The clinical tests of digoxin as an anticancer drug, alone or in combination with chemotherapeutic drug were reported in recent years (Menger et al., 2013; Frankel et al., 2017; Huang et al., 2018). However, the anticancer mechanism of digoxin has not yet been fully elucidated.

Inhibition of Na^+^/K^+^-ATPase and topoisomerase (Beheshti Zavareh et al., 2008), alterations of Ca^2+^ signaling (Raynal et al., 2016), as well as inhibition of HIF-1α synthesis (Zhang et al., 2008; Gayed et al., 2012) were reported to play roles in the anticancer effects of digoxin. And signaling pathways such as Src/MAPK (Wang et al., 2009), Akt/mTOR (Zhang et al., 2017), NF-κB (Wang et al., 2017) and Nrf2 (Zhou et al., 2019) were also known to be involved. Remarkably, digoxin was recently reported to inhibit DNA double strand break repair as a novel anticancer mechanism (Surovtseva et al., 2016).

DNA double strand break (DSB) repair and single strand break (SSB) repair pathways are part of the cellular DNA damage repair (DDR) network (Brown et al., 2017), crucial to maintain the survival and proliferation of cancer cells. So far, drug inhibition of DDR has paved the road to new therapeutic approaches in oncology (Carrassa and Damia, 2017; Nickoloff et al., 2017). The first FDA-approved, DDR-targeted cancer therapeutic drug is olaparib, an inhibitor of poly (ADP-ribose) polymerases (PARP) for the treatment of cancers with inherited BRCA1 or 2 mutations by inducing the synthetic lethality (Lord and Ashworth, 2017). Currently, there are various inhibitors of DDR in preclinical and clinical development for cancer therapy.

Digoxin has been proved to inhibit DNA DSB repair (Surovtseva et al., 2016), and strongly potentiate the induction of DNA damage by ionizing radiation (IR) treatment through reducing the expression of DDR proteins in radio-resistant A549 cells (Lee et al., 2017). However, the effect of digoxin on SSB repair has not been reported.

On the other hand, chemotherapeutic drugs such as adriamycin remain to be cornerstones in cancer treatment. However, clinical use of adriamycin is limited by cardiotoxicity. Recently, Acovenoside A, another kind of cardiac glycosides was reported to prevent adriamycin-induced cardiotoxicity in mice (Ezzat et al., 2016). Digoxin was also found to prevent chronic adriamycin cardiotoxicity clinically (Whittaker and Al-Ismail, 1984). However, no experimental evidence for the therapeutic potential of digoxin in adriamycin-mediated cardiotoxicity has been reported. Herein, we demonstrated that digoxin exhibited antitumor effects on human non-small cell lung cancer cells by inhibiting both DNA DSB and SSB repair. Furthermore, combination with adriamycin showed the synergistic anticancer effects both *in vitro* and *in vivo*. Moreover, digoxin reduced adriamycin-induced cardiotoxicity.

## Materials and Methods

### Reagents

Digoxin was purchased from Aladdin (London, ON, Canada). Adriamycin was from Dalian Meilun Biological Product Factory (Dalian, LN, China). Anti-RPA32/RPA2, anti-Histone H2AX (phospho S139) and anti-XRCC1 were obtained from Abcam (Cambridge, MA, USA). Anti-β-actin, anti-RAD51, anti-mouse and anti-rabbit HRP-conjugated secondary antibodies were obtained from Cell Signaling Technology (Danvers, MA, USA). 2′, 7′-Dichloro fluorescein diacetate (DCFH-DA) were purchased from Sigma-Aldrich (St. Louis, MO, USA). 3-(4, 5-dimethyl-2-thiazolyl)-2, 5-diphenyl-2-H-tetrazolium bromide (MTT) was from Amresco (Solon, OH, USA).

### Cell Culture

Human non-small cell lung cancer A549 and H1299 cells were obtained from Cell Resource Center, Peking Union Medical College (Beijing, China). The cells were cultured in RPMI 1640 medium supplemented with 10% fetal bovine serum, 10 μg/mL of streptomycin, and 100 U/mL of penicillin at 37°C in a humidified atmosphere containing 5% CO_2_. All human cell lines have been authenticated using short-tandem repeats (STR) profiling within the last three years. All experiments were performed with mycoplasma-free cells.

### Cell Viability Assay

The MTT assay was performed to assess cell viability as reported by us previously (Peng et al., 2018). Briefly, two hundred microliters of cells (3×10^4^ cells/mL or 2×10^4^ cells/mL) were cultured in a 96-well plate treated with various concentrations of digoxin or adriamycin for 24 h or 48 h. After addition of MTT (5 mg/mL) to each well, the cells were further incubated for 4 h. The produced formazan blue was dissolved with dimethyl sulfoxide (DMSO), and the absorbance was measured at 490 nm using microplate reader (iMark, Bio-Rad). 

### Comet Assay

Comet assay was carried out to evaluate the DNA damage as reported (May et al., 2018). Briefly, 6 × 10^4^ cells exposed to digoxin (0, 0.025, 0.05, and 0.1 μM) for 24 h were suspended in 0.65% low melting agarose, and then spread on the 1% normal melting agarose-coated slides. The slides were left at 4°C to solidify the low melting agarose. After that the slides were immersed in lysis solution (2.5 M NaCl, 100 mM Na_2_EDTA, 10 mM Tris base, 1% Triton-X 100, and 10% DMSO) overnight at 4°C. Subsequently, the slides were transferred to an electrophoretic box containing 300 mM NaOH and 1 mM Na_2_EDTA (pH > 13) for 30 min at 4°C before electrophoresis for 30 min at 300 mA, 25 V at 4°C. Thereafter, the slides were rinsed with neutralizing buffer (0.4 M Tris HCl, pH = 7.5) for three times, buried in dehydration ethanol for 30 min, then stained with 10 × GoldView I solution. Finally, the slides were examined with laser scanning confocal microscope (FV1000, Olympus), equipped with an excitation filter of 230 nm or 490 nm. Randomly chosen cells were scored visually by CASP image-analysis program. Tail intensities in percentages (= % DNA in tail) are expressed as the mean and corresponding standard deviations of at least three independent experiments.

### Measurement of Intracellular ROS Generation

Intracellular ROS level was measured using DCFH-DA ﬂuorescent probe as we reported (Tang et al., 2014). After exposure to digoxin for 24 h, A549 and H1299 cells were incubated with 10 μM of DCFH-DA for 30 min at 37°C, respectively. The resulting ﬂuorescent intensity at 488 and 530 nm was measured using flow cytometer (Accuri C6, BD Biosciences).

### Western Blot Analysis

Western blot analysis was carried out as described by us previously (Zhang et al., 2018). Lysates of cells treated with indicated digoxin, adriamycin or DMSO (0.1%) as control were prepared. Proteins in the cell lysates were separated by sodium dodecyl sulfate-polyacrylamide gel electrophoresis (SDS-PAGE), and then transferred onto polyvinylidene fluoride membranes (Millipore, Billerica, MA, USA). After being blocked, the membranes were incubated with each primary antibody, and then the respective secondary antibody. Signals from the bound antibodies were detected using ChemiDoc XRS+System (Bio-Rad), and quantified using the software of ImageJ with the respective β-actin signal as background.

### Immunofluorescence

Cells were plated onto coverslips and incubated for 24 h with digoxin (0, 0.05, 0.15 and 0.2 μM) as indicated. Coverslips were rinsed in phosphate-buffered saline (PBS) at 37°C and fixed in 3% paraformaldehyde, 0.1% Triton X-100 for 20 min at room temperature, then incubated with primary antibody for 12 h at 4°C, followed by 1 h incubation at room temperature with the appropriate secondary antibody. Coverslips were washed in PBS. Cell nuclei were stained with 1 mg/mL Hoechst 33342 (blue). Images were obtained with an inverted confocal microscope (LSM 800, Zeiss) and analyzed by Adobe PhotoShop.

### Synergism Assay

Synergism assay was conducted to analyze whether combination of digoxin with adriamycin can achieve an improved therapeutic effect. Cell growth inhibition activities of each single drug and the combination at three different ratios (IC50_DIG_: IC50_ADR_, 1/2IC50_DIG_: IC50_ADR_, 1/4IC50_DIG_: IC50_ADR_) were tested, respectively. The combination index (CI) was calculated using CalcuSyn software according to the method of Chou and Talalay. CI > 1 indicates antagonism, CI = 1 indicates additivity, CI < 1 indicates synergism. All experiments were carried out in triplicate.

### Zebraﬁsh A549 Xenograft Model

Zebrafish were raised and maintained at 28°C in E3 media as reported (Westerfield, 1995). Embryos at 48 h post-fertilization (hpf) were anesthetized using 1.2 mM of tricaine (Sigma-Aldrich, St. Louis, MO, USA), moved onto a modiﬁed agarose gel mold for tumor cell microinjection. Before injection, A549 cells were labeled *in vitro* with 5 μM of DiO. Approximately 100 DiO-labeled A549 cells in a volume of 50 nL were injected into the perivitelline space of each embryo using a pneumatic picopump (WPI) and a micromanipulator (X221942H, Nikon). Injected embryos were transferred to a 24-well plate containing drug in 2 mL E3 media and incubated at 32°C for 48 h. Embryos were imaged with fluorescence microscope (IX71, Olympus).

### Nude Mouse A549 Xenograft Model

BALB/c nude mice (16–18 g, purchased from Charles River, Beijing, China, certificate no. SCXK (Jing) 2016-0006) were used. The committee for animal use at Beijing Institute of Pharmacology and Toxicology approved all experimental procedures. A549 Cells were harvested by trypsinization, rinsed with PBS, and resuspended at 1 × 10^7^ cells per milliliter in PBS, and then subcutaneously injected into the right flank of BALB/c nude mice. When the tumor size reached approximately 100 mm^3^, 20 mice were separated into four groups and respective treatments were given. Group I (Control): saline only; Group II: 1.0 mg/kg/d digoxin, *i.p.*; Group III: 2.0 mg/kg/3d adriamycin, *i.p.*; Group IV: 1.0 mg/kg/d digoxin + 2.0 mg/kg/3d adriamycin, *i.p.* Tumor volume was measured once every two days (calculated as volume = shortest diameter^2^ × longest diameter/2). Body weight was recorded once every two days. After 14 days, the mice were sacrificed, and the tumors and hearts were excised, weighed out, and stored at −80°C or fixed in 4% paraformaldehyde until further analysis.

### Histological and Immunohistochemical Analyses

Samples of tumors and hearts were dehydrated, paraffin embedded, and sectioned into 4 μm thick slices on a sliding microtome (HM325, Thermo). For immunohistochemistry, slices were autoclaved at 120°C for 5 min in citrate buffer (pH = 6), and quickly cooled with distilled water. Endogenous peroxidase activity was blocked by incubating the samples in PBS containing 3% H_2_O_2_ for 10 min. Sections were saturated in PBS supplemented with 2.5% goat serum for 30 min at room temperature, then incubated with primary antibodies for 1 h at room temperature. Then the sections were further exposed to biotinylated secondary antibodies (DAKO, Glostrup, DK) and DAB (Thermo, Waltham, MA, USA) for about 5 min. Finally, counterstaining was performed with hematoxylin. The other sections were stained with hematoxylin and eosin. Slices were visualized under a fluorescent scan system (Aperio Scanscope). The percentage of Ki-67, γH2AX-positive cells were quantified by counting brown-stained cells at five arbitrarily selected fields from each tumor at 400× magnification.

### Statistical Analysis

Data are presented as mean ± SD, representative of at least three independent experiments. One-way ANOVA was used to determine the statistical significance of differences between groups. All statistical analyses were performed using SPSS software, and differences were considered statistically significant when the *P*-value was less than 0.05.

## Results

### Digoxin Inhibited Cell Viability, Increased DNA Damage and ROS Production of NSCLC A549 and H1299 Cells

**Figure 1:**
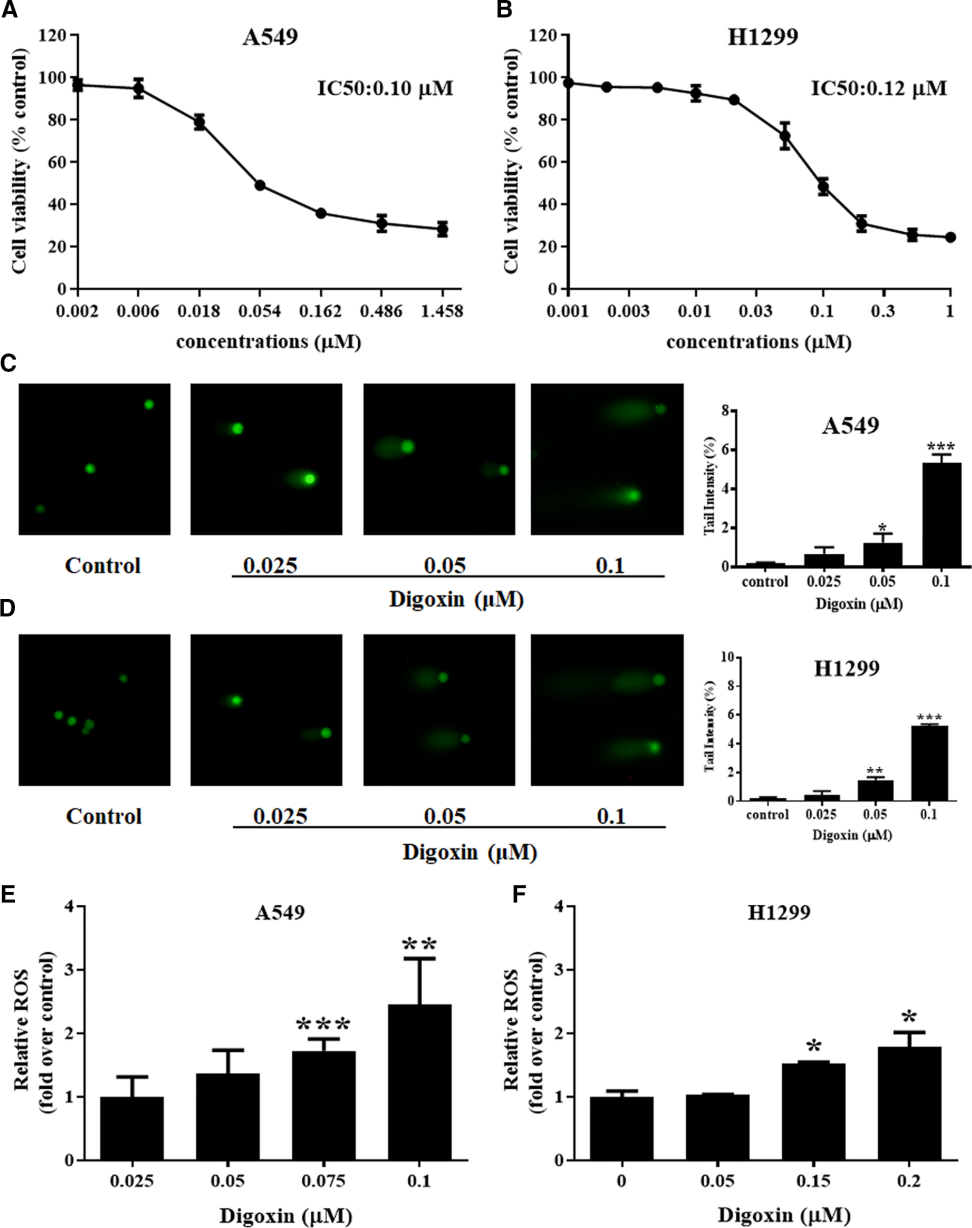
Digoxin inhibited cell viability, increased DNA damage and ROS production of A549 and H1299 cells. **(A, B)** A549 and H1299 cells were treated with various concentrations of digoxin for 24 h. The cell viability was determined using MTT assay. **(C, D)** The potential of digoxin to cause DNA damage, expressed as % tail intensity, was determined on A549 and H1299 cells using the comet assay. **(E, F)** A549 and H1299 cells were stained by DCFH-DA (10 μM) for 30 min. The ROS levels were determined by flow cytometer, and expressed as fold over that in untreated cells. Data are mean ± SD (*n* = 3), representative of 3 independent experiments. *: *p <* 0.05, **: *p <* 0.01, ***: *p <* 0.001, compared with control.

We first examined the antiproliferative effects of digoxin on A549 and H1299 cells after treatment for 24 h by using MTT assay. As shown in **Figures 1A, B**, digoxin inhibited proliferation of A549 and H1299 cells in a dose-dependent manner, with IC50 values of 0.10 and 0.12 µM for A549 and H1299 cells, respectively. The ability of digoxin to induce DNA damage in the NSCLC cells was determined with comet assay. **Figures 1C, D** showed the DNA damage as measured by percentage tail intensity in A549 and H1299 cells after exposure to different concentrations of digoxin for 24 h, respectively. The tail intensity of A549 and H1299 cells increased significantly in a dose-dependent manner, indicating that digoxin could significantly induce DNA damage in NSCLC cells. ROS can induce DNA damage and affect the DDR. Thus we investigated the effect of digoxin on ROS generation. As shown in **Figures 1E, F**, digoxin increased the ROS level in A549 and H1299 cells in a dose-dependent manner.

### Digoxin Inhibited the DSB and SSB Repair in NSCLC A549 and H1299 Cells

**Figure 2:**
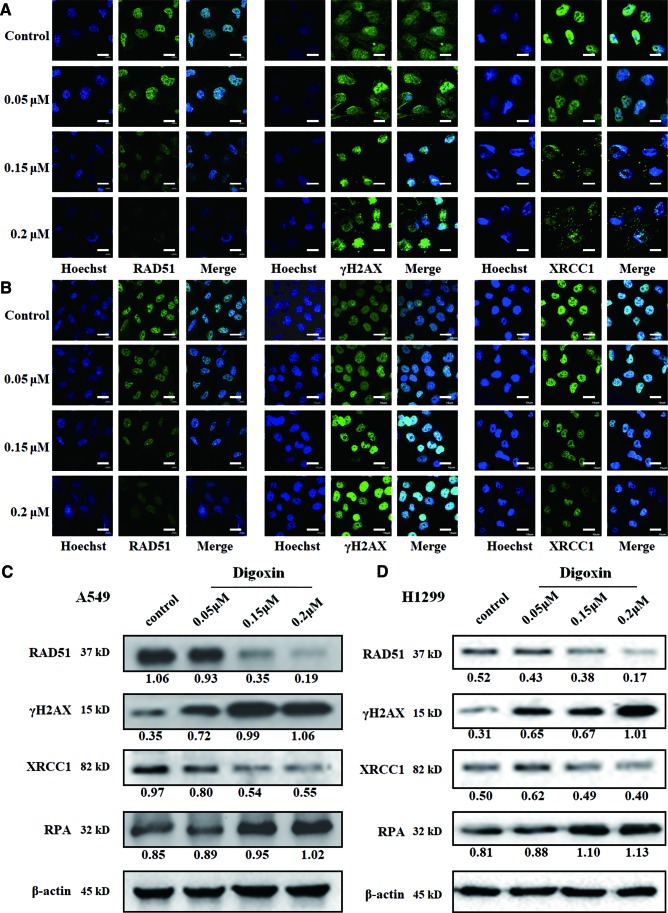
Digoxin inhibited DNA DSB and SSB repair in A549 and H1299 cells. **(A, B)** Immunofluorescence staining of RAD51, γH2AX, and XRCC1 in A549 and H1299 cells. Scale bars: 20 µm. **(C, D)** The expression levels of RAD51, γH2AX, XRCC1 and RPA were determined in A549 and H1299 cells by western blot using the indicated antibodies.

To evaluate the role of digoxin in DNA damage repair, we first determined whether digoxin affected DNA DSB repair using the immunofluorescence and western blot. The γH2AX and RPA were used to mark the damaged DNA double strand and single strand, respectively. Meanwhile RAD51 and XRCC1 were used to mark the repair of the damaged DNA double strand and single strand, respectively. Both A549 and H1299 cells treated with digoxin showed significantly reduced expression of RAD51 in a dose-dependent manner. Accordingly, enhanced γH2AX levels in a dose-dependent manner were observed (**Figure 2**). These results indicated that digoxin inhibited DSB repair in NSCLC.

We further investigated whether digoxin affected the SSB repair. XRCC1 was diffused in control cancer cells, the resolution was incomplete and XRCC1 foci were observed in digoxin-treated cells. Meanwhile, the expression of XRCC1 was decreased, but that of RPA was increased. These results indicated that digoxin inhibited DNA SSB repair in NSCLC.

### The Synergistic Antiproliferative Effects of Digoxin and Adriamycin on NSCLC A549 and H1299 Cells

We also investigated the antiproliferative effects of digoxin on A549 and H1299 cells in combination with adriamycin. The cells were cultured in the presence of digoxin, adriamycin or both compounds at 37°C for 48 h. As shown in **Figures S1A** and **S2A**, digoxin and adriamycin alone inhibited the proliferation of A549 and H1299 cells dose-dependently, with the IC50 values to be 0.037 and 0.14 µM (A549), and 0.054 and 0.95 µM (H1299), respectively. Then, the synergistic effect was analyzed by using three constant ratios of two drugs (IC50_DIG_: IC50_ADR_, 1/2IC50_DIG_ : IC50_ADR_, 1/4IC50_DIG_ : IC50_ADR_) at a series of concentration combinations (20%, 40%, 60%, 80%, 100% of the IC50 values of each drug). As shown in **Figures S1B–D** and **S2B–D**, the panel of 1/2IC50_DIG_:IC50_ADR_ and IC50_DIG_: IC50_ADR_ exerted synergistic antiproliferative effect on A549 and H1299 cells, respectively. The combination indexes (CI) at ED50, ED75 and ED90 were calculated and shown in **Tables 1** and **2**.

**Table 1:** Combination indexes (CI) of digoxin and adriamycin for A549 cells.

Drug or drug combination	r	CI values		
		ED_50_	ED_75_	ED_90_
DIG	0.978	—	—	—
ADR	0.995	—	—	—
DIG+ADR(IC_50_: IC_50_)	0.996	0.581	1.124	2.860
DIG+ADR(1/2×IC_50_:IC_50_)	0.994	0.444	0.294	0.393
DIG+ADR(1/4×IC_50_: IC_50_)	0.997	1.289	12.52	355.6

**Table 2:** Combination indexes (CI) of digoxin and adriamycin for H1299 cells.

Drug or drug combination	r	CI values		
		ED_50_	ED_75_	ED_90_
DIG	0.979	—	—	—
ADR	0.993	—	—	—
DIG+ADR(IC_50_:IC_50_)	0.944	0.988	0.577	0.699
DIG+ADR(1/2×IC_50_:IC_50_)	0.996	2.207	4.491	10.319
DIG+ADR(1/4×IC_50_:IC_50_)	0.963	0.321	1.367	16.151

### Combination of Adriamycin With Digoxin Further Enhanced DNA Damage and Reduced SSB Repair

**Figure 3:**
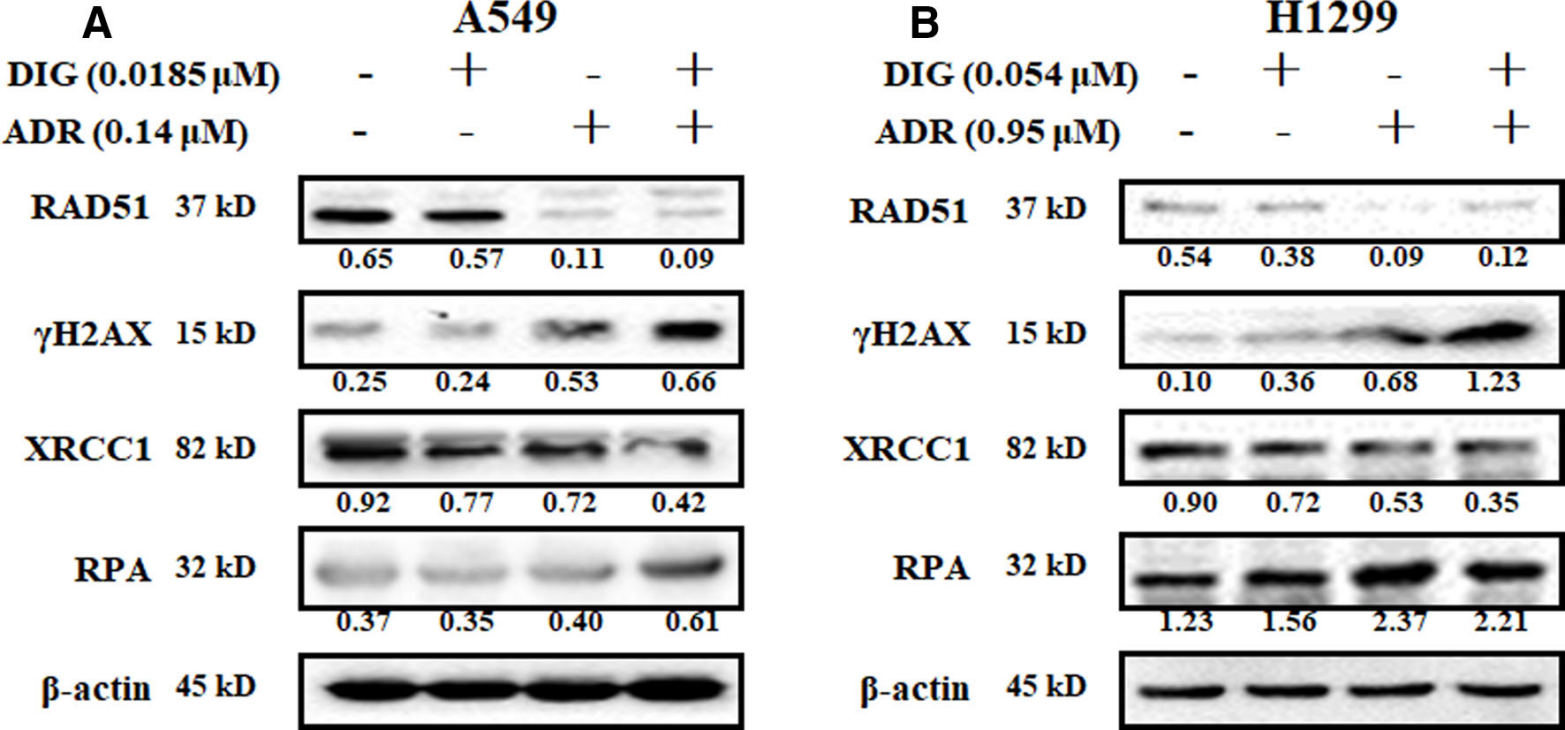
The effect of the combination of digoxin and adriamycin on DNA damage-related molecules. **(A**, **B)** A549 and H1299 cells were incubated with indicated digoxin or adriamycin alone or in combination for 48 h. The cells were harvested, and the cell lysates were prepared to be available for western blot analysis for RAD51, γH2AX, XRCC1 and RPA expression levels.

We further investigated the effect of digoxin in combination with adriamycin on DNA damage related proteins. **Figure 3** showed that adriamycin alone could remarkably reduce the expression of RAD51 and increase the expression of γH2AX in A549 and H1299 cells. When combined with digoxin, the expression of γH2AX was increased. On the other hand, adriamycin alone slightly inhibited the expression of XRCC1 on both A549 and H1299 cells. When combined with digoxin, the expression of XRCC1 was further reduced, suggesting the DNA SSB repair might be inhibited by the combination.

### Digoxin Alone or in Combination With Adriamycin Inhibited A549 Tumor Growth in Zebraﬁsh Model

**Figure 4:**
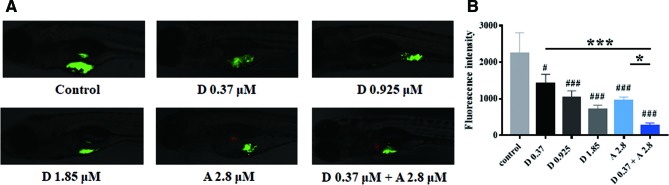
Digoxin alone or co-administration with adriamycin inhibited tumor growth in zebrafish A549 xenograft model. **(A)** A549 cells were labeled with DiO (green fluorescence), and injected into each embryo. The injected embryos were transferred to a 24-well plate containing drug and incubated for 48 h. The embryos were imaged with fluorescence microscope to detect tumor growth. **(B)** Quantification of tumor size as fluorescence intensity. Data are expressed as means ± SD (n = 5). ^*^: *p <* 0.05, ^***^: *p <* 0.001, compared with each other; ^#^: *p <* 0.05, ^###^: *p <* 0.001, compared with control.

We then evaluated the *in vivo* antitumor efficacy of digoxin alone or in combination with adriamycin by using the zebraﬁsh tumor model. As indicated in **Figure 4**, digoxin alone reduced the growth and dissemination of A549 cells in zebrafish in a dose-dependent manner. Combination with adriamycin enhanced the antitumor effect significantly.  

### Digoxin Alone or in Combination With Adriamycin Inhibited A549 Tumor Growth in Nude Mouse Xenograft Model

**Figure 5:**
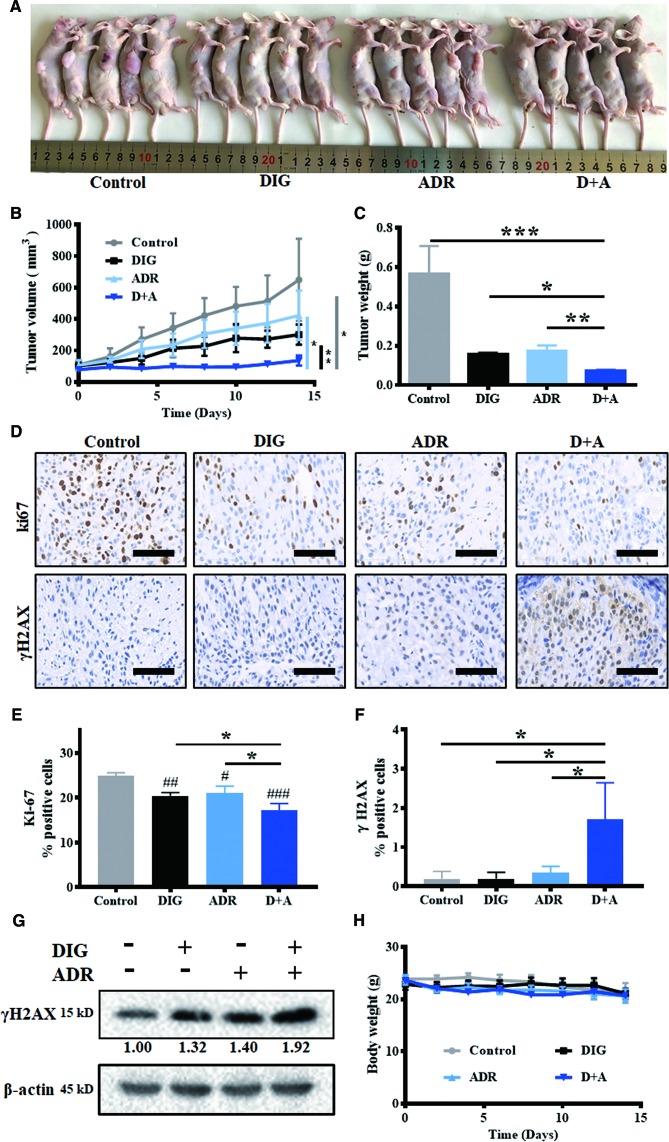
Digoxin alone or co-administration with adriamycin inhibited tumor growth in nude mouse A549 xenograft model. **(A)** Photographs of control, DIG, ADR, and D+A-treated mice. **(B)** Tumor volume was measured once every 2 days and calculated based on the formula of tumor volume = shortest diameter^2^ × longest diameter/2. **(C)** Tumor weight was measured at the end of experiment. **(D)** Tumor tissue samples were immunohistochemically analyzed for Ki67, γH2AX-positive cells. Scale bars: 60 µm. **(E, F)** Quantification for Ki67 and γH2AX-postive cells from 5 mice in each group. **(G)** Western blot analysis for γH2AX expression in tumor tissues. **(H)** Body weight changes of the mice in each group for 14 days. Data are expressed as means ± SD (*n* = 5). *: *p <* 0.05, **: *p <* 0.01, ***: *p <* 0.001, compared with each other; ^#^: *p <* 0.05, ^##^: *p* < 0.01, ^###^: *p* < 0.001, compared with control.

To further evaluate the *in vivo* antitumor efficacy of digoxin alone or in combination with adriamycin, nude mouse A549 xenograft model was constructed. As shown in **Figures 5A–C**, digoxin (1.0 mg/kg/d) alone significantly inhibited A549 tumor growth. When combined with adriamycin (2.0 mg/kg/3d), the effect was enhanced obviously. Meanwhile, immunostaining and western blot experiment indicated that the expression of Ki67 was decreased, and γH2AX was increased (**Figures 5D–G**), suggesting that the enhanced antitumor effect might be attributed to the increased DNA damage by the combination. In addition, the body weight was not obviously reduced by each treatment, suggesting the low systemic toxicity of either drug alone or in combination (**Figure 5H**).

### Digoxin Reduced the Cardiotoxicity Caused by Adriamycin

**Figure 6:**
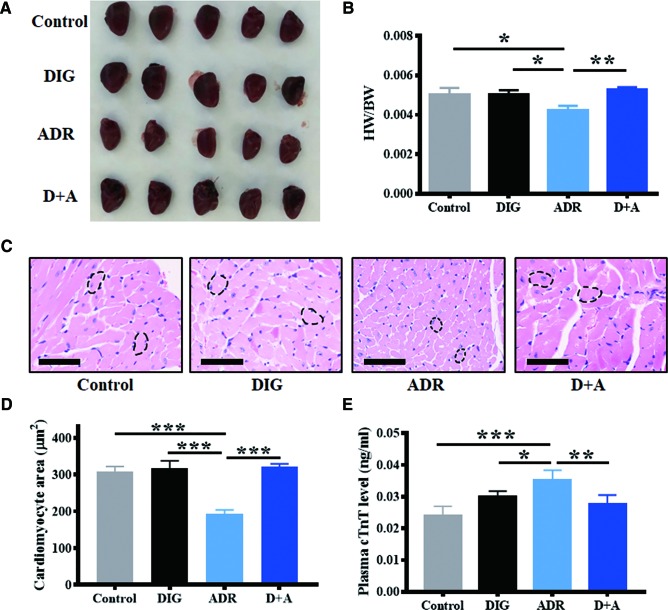
Digoxin protects from adriamycin induced cardiotoxicity. **(A)** Representative hearts from A549 xenograft nude mice 14 days after treatment without or with adriamycin and digoxin alone or in combination. **(B)** Evaluation of heart weight/body weight (HW/BW) of the xenograft mice in each group at the end of experiment. **(C)** Heart samples were H&E stained to detect cardiomyocyte. Scale bars: 60 µm. **(D)** Quantification for cardiomyocyte area from 5 mice in each group. **(E)** Plasma troponin T (TnT) level of the xenograft mice in each group at the end of experiment. Data are expressed as means ± SD (*n* = 5). ^*^: *p* < 0.05, ^**^: *p* < 0.01, ^***^: *p* < 0.001, compared with each other.

The clinical use of adriamycin is hampered by cardiotoxicity. To investigate whether digoxin can ameliorate the cardiotoxicity caused by adriamycin, we determined the heart size, cardiomyocyte size as well as the plasma concentration of troponin T, which is known as a cardiac injury marker. As shown in **Figure 6**, adriamycin alone significantly reduced the heart size and cardiomyocyte size, increased plasma concentrations of troponin T. However, co-treatment with digoxin obviously blocked the effect, suggesting digoxin could reverse the reduction of heart weight/body weight ratio (HW/BW) by adriamycin. In addition, there are no differences among the digoxin, digoxin combined with adriamycin, and control group in HW/BW or cardiomyocyte size after 14 days treatment, suggesting the low cardiotoxicity of either digoxin alone or in combination with adriamycin.

## Discussion

In this paper, we reported digoxin exhibited antitumor activities on NSCLC A549 and H1299 cells. Treatment with digoxin promoted DNA damage, inhibited both DNA DSB repair and SSB repair. Combination with adriamycin produced a synergistic antiproliferative effect. Furthermore, digoxin alone or in combination with adriamycin exerted favorable *in vivo* antitumor efficacy on A549 zebrafish model and nude mouse model. Interestingly, co-treatment with digoxin not only enhanced the antineoplastic effect of adriamycin, but also reduced the cardiotoxicity.

Digoxin was used as a cardioprotectant drug as an inhibitor of Na^+^/K^+^-ATPase. On the other hand, it was frequently reported to show anticancer effects (Platz et al., 2011; Menger et al., 2012; Huang et al., 2018), while the anticancer mechanism remained unclear. Some reports suggest that digoxin exert anticancer effects by inhibiting the Na^+^/K^+^-ATPase which is overexpressed in the membrane of some cancer cells, and therefore inducing multiple cell death modalities such as apoptosis, autophagy, anoikis, and immunogenic cell death (Wang et al., 2015; Schneider et al., 2017). However, since the EC50 values for inhibiting Na, K-ATPase are 0.52~1.34 μM (Mijatovic et al., 2007), much higher than its antitumor IC50 values (0.10 and 0.12 µM for A549 and H1299) in our study, we predict that the antitumor activity might not be attributed to its inhibition against Na^+^/K^+^-ATPase.

HIF-1 is also known to be a critical target of digoxin for cancer therapy. Digoxin was reported to inhibit HIF-1α protein synthesis and expression of HIF-1 target genes in cancer cells, and therefore block primary tumor growth, vascularization, invasion, and metastasis (Semenza, 2012; Samanta et al., 2014).

Recently, digoxin was reported to act as radio-sensitizer, by inhibiting DSB repair and therefore potentiating DNA damage caused by IR treatment. The report indicated that digoxin could elevate the expression of γH2AX, promote the formation of γH2AX foci, and reduce the expression of RAD51 (Lu et al., 2014; Surovtseva et al., 2016; Lee et al., 2017), consistent with our finding in this study. Besides, we found that digoxin reduced the expression of XRCC1 and increased the expression of RPA. Since RPA plays an important role in protecting fragile single stranded DNA intermediates, and XRCC1 is a SSB repair effector protein (Pilié et al., 2019), our result suggested that the SSB repair was also inhibited by digoxin. Therefore, we demonstrated digoxin could potentiate the DNA damage of cancer cells through inhibition of both DNA DSB and SSB repair. In addition, we also found that ROS generation was increased after digoxin treatment. Since accumulation of ROS might lead to DNA damage, enhancement of ROS production might also contribute to the DNA damage induced by digoxin.

Since drug combination is expected to enhance efficacy and reduce side effects by decreasing the dose of each drug, and the narrow therapeutic window hampered the clinic application of digoxin, we investigated the antitumor effects of digoxin in combination with a chemotherapeutic drug adriamycin. The growth inhibition of cancer cells by the chemotherapeutic drug could be reliably determined at 2 to 4 days of drug exposure (Monks et al., 1991), thus the *in vitro* synergism assay was carried out after 48 h incubation. As a result, digoxin enhanced the antitumor effects of adriamycin *in vitro* and *in vivo*. Interestingly, co-treatment with digoxin obviously reduced the cardiotoxicity caused by adriamycin, suggesting that the combination not only resulted in enhanced efficacy but also reduced toxicity. However, the mechanism of cardiac glycoside to prevent Adriamycin induced cardiotoxicity is still unclear. It was reported that the cardenolide glycoside Acovenoside A protected from adriamycin-induced cardiotoxicity in mice by inhibiting oxidative stress and inflammation (Ezzat et al., 2016). Besides, ophiopogonin D, another steroidal glycoside, attenuated adriamycin-induced autophagy *in vitro* and *in vivo* through inhibiting JNK and ERK pathways (Zhang et al., 2015).

Our work indicated that digoxin enhanced the anticancer effect on non-small cell lung cancer while reduced the cardiotoxicity of adriamycin, suggesting the combination might affect DNA damage in a cell context-dependent manner, which was demonstrated in **Figure S3**. This is the first report about the antitumor efficacy of combination of digoxin and adriamycin, with reduced cardiotoxicity. In addition, we firstly demonstrated that digoxin exerted antitumor effects by inhibiting DNA SSB repair, besides inhibiting the DSB repair and inducing the generation of ROS.

Although the results presented here are encouraging, the concentrations of digoxin utilized in this study are still higher than those tolerable in the circulation (1–2 ng/mL) (Pashazadeh-Panahi and Hasanzadeh, 2020). Therefore, further work is required to enhance the concentration of digoxin in tumor while reducing the systemic toxicity. Actually, we have just started such work by using nanotechnology. Our current work in this paper represents proof of concept studies that digoxin or its derivatives might have the desired anticancer effects on NSCLC.

Digoxin exerted anticancer activity on NSCLC through inhibiting DNA DSB and SSB repair and promoting ROS generation. Combination with adriamycin showed synergistic effects *in vitro*. Compared with adriamycin alone, cotreatment with digoxin led to an enhanced antitumor efficacy and a reduced cardiotoxicity, suggesting that digoxin has the potential to become an antitumor drug in combination with adriamycin for therapy of human NSCLC, while further investigation is needed.

## Data Availability Statement

All datasets generated for this study are included in the article/**Supplementary Material**.

## Ethics Statement

The committee for animal use at Beijing Institute of Pharmacology and Toxicology approved all experimental procedures.

## Author Contributions

YW acquired the *in vitro* data. QM, SZ, and HL acquired the *in vivo* data. BZ, WW, and PL analyzed the *in vitro* data. BD analyzed the *in vivo* data. YW and ZZ drafted the article. ZZ, YZ, and DK designed the experiments. YZ and DK edited the article.

## Funding

This study was supported by grants from National Natural Science Foundation of China (81673464), Major Project of Tianjin for New Drug Development (17ZXXYSY00050), the Science & Technology Development Fund of Tianjin Education Commission for Higher Education (2017KJ230), the Natural Science Foundation for Young Scientists of Tianjin (18JCQNJC83500), and Natural Science Foundation of Tianjin Science and Technology (15JCYBJC27500).

## Conflict of Interest

The authors declare that the research was conducted in the absence of any commercial or financial relationships that could be construed as a potential conflict of interest.
