# Supplementary material for: Digoxin Enhances the Anticancer Effect on Non-Small Cell Lung Cancer While Reducing the Cardiotoxicity of Adriamycin
Source: Front Pharmacol. 2020 Feb 28;11:186. doi: 10.3389/fphar.2020.00186 (PMC7059749; doi:10.3389/fphar.2020.00186)
Supplement: Supplementary file 1 [file DataSheet_1.docx]

**Digoxin enhances the anticancer effect on non-small cell lung cancer while reducing the cardiotoxicity of adriamycin**

**Yingying Wang^1^**^#^**, Qian Ma^1,^** **^2^**^#^**, Shaolu Zhang^1,2^, Hongyan Liu^2^, Baoquan Zhao^2^, Bo Du^3^, Wei Wang^4^, Peng Lin^4^, Zhe Zhang^1*^, Yuxu Zhong^2*^, Dexin Kong^1*^**

^1^Tianjin Key Laboratory on Technologies Enabling Development of Clinical Therapeutics and Diagnostics, School of Pharmaceutical Sciences, Tianjin Medical University, Tianjin 300070, China

^2^State Key Laboratory of Toxicology and Medical Countermeasures, Beijing Institute of Pharmacology and Toxicology, Beijing 100850, China

^3^Tianjin Key Laboratory of Biomedical Materials, Institute of Biomedical Engineering, Chinese Academy of Medical Sciences & Peking Union Medical College, Tianjin 300192, China

^4^Department of Otorhinolaryngology Head and Neck, Institute of Otorhinolaryngology, Tianjin First Central Hospital, Tianjin 300192, China

*** Correspondence:**Corresponding Author
zhangzhe@tmu.edu.cn; yuxuzhong2008@aliyun.com; kongdexin@tmu.edu.cn

**
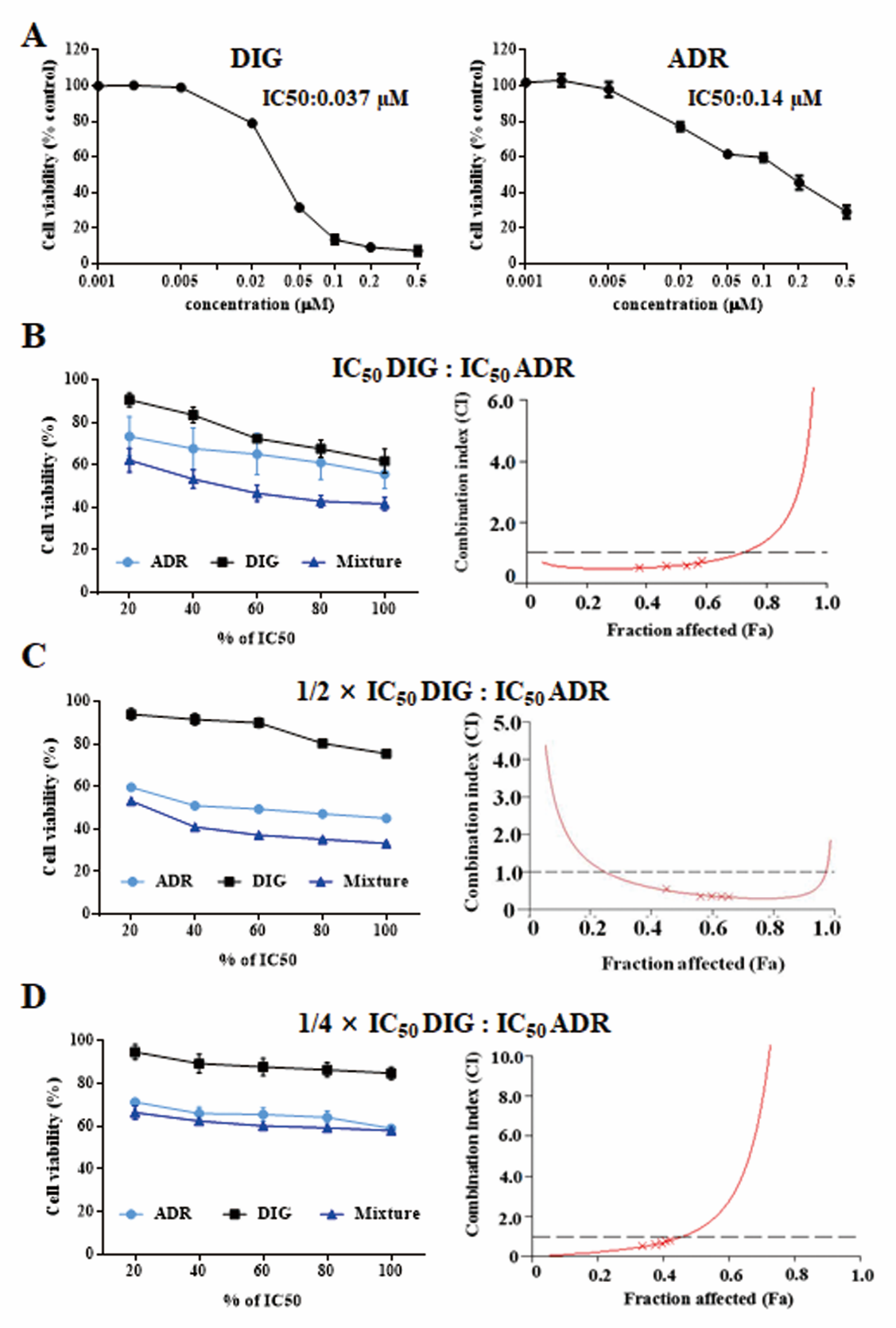
**

Fig. S1 Combination of digoxin with adriamycin led to synergistic antiproliferative effect on A549 cells. A549 cells were treated with digoxin and adriamycin as a single agent or in combination for 48 h. (A) The growth inhibitory effect of digoxin and adriamycin as a single agent on A549 cells. Three fixed ratios were used to investigate the combinational effect: IC50_DIG_: IC50_ADR_ (B); 1/2IC50_DIG_: IC50_ADR_ (C), 1/4IC50_DIG_: IC50_ADR_ (D). The cell viability after treatment was measured by MTT assay (left). Combinational effect was analyzed using CalcuSyn software and the resulting CI-Fa plots are shown (right). Data are mean ± SD (*n* = 3), representative of 3 independent experiments.


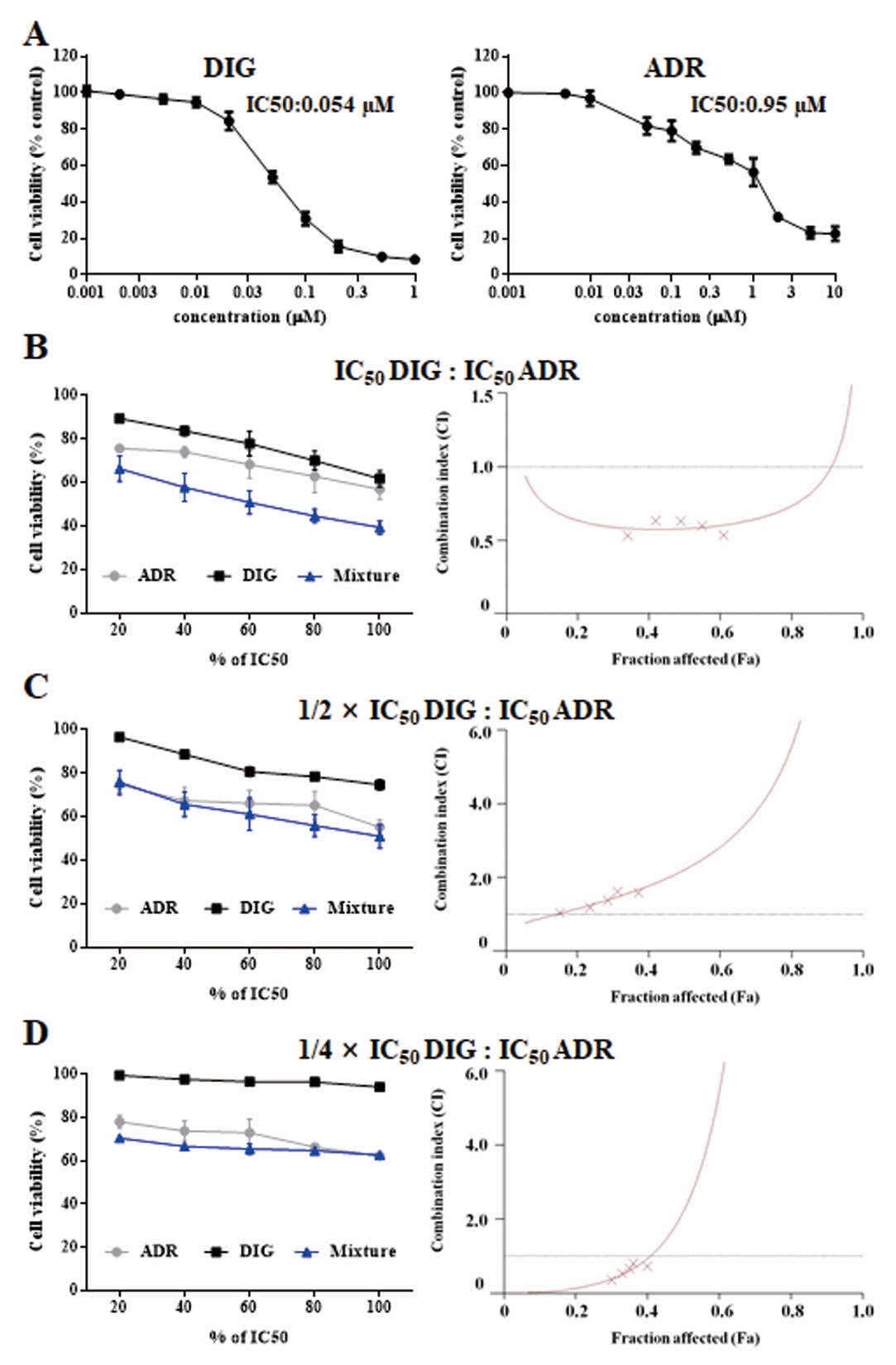


Fig. S2 Combination of digoxin with adriamycin led to synergistic antiproliferative effect on H1299 cells. H1299 cells were treated with digoxin and adriamycin as a single agent or in combination for 48 h. (A) The growth inhibitory effect of digoxin and adriamycin as a single agent on H1299 cells. Three fixed ratios were used to investigate the combinational effect: IC50_DIG_: IC50_ADR_ (B); 1/2IC50_DIG_: IC50_ADR_ (C), 1/4IC50_DIG_: IC50_ADR_ (D). The cell viability after treatment was measured by MTT assay (left). Combinational effect was analyzed using CalcuSyn software and the resulting CI-Fa plots are shown (right). Data are mean ± SD (*n* = 3), representative of 3 independent experiments.


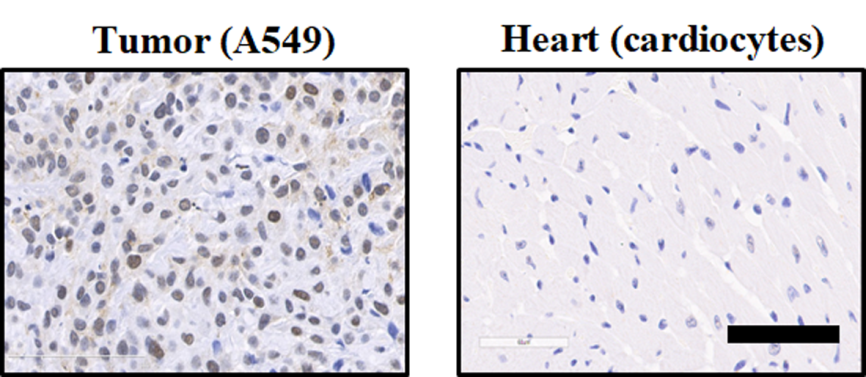


FigS. 3 Digoxin co-administration with Adriamycin inhibited the DNA damage repair on a cell context-dependent manner. Tumor and heart were immunohistochemically analyzed for γH2AX-positive cells. Scale bars: 60 µm.
